# Separating Teeth by Mechanical Pressure

**Published:** 1860-01

**Authors:** J. Canning Allen


### SEPARATING TEETH BY MECHANICAL PRESSURE.

BY J. CANNING ALLEN.

It is well known that the object of this operation is generally to obtain space to fill properly, or for the perfect removal of superficial caries; I shall, therefore, without any further premising, proceed at once to speak of the evils arising from improperly and imprudently separating teeth in this manner.

Forcing teeth apart by mechanical pressure moves them from their original positions and starts them in their sockets. Now, though the simple operatiou of merely moving teeth in their sockets may seem to be of but little consequence, yet, to the observing practitioner, who has so many opportunities of seeing the pernicious effects resulting from this operation when mal-performed, the manne: of its proper execution must be a matter of no trifling weight. How often do we hear persons remark, who have had their teeth separated in this way, that they will never have it repeated, giving as a reason that ever since the completion of the operation these organs have been continually getting sore and painful. I am compelled to acknowledge that in many cases this statement of the matter is but too true.

Are these consequences *necessary evils* following this operation? In my humble opinion they are not, but occur only (in the majority of cases) where the work has been injudiciously carried on. I now purpose speaking, with as much brevity as possible, of the different articles used by our profession for separating teeth by mechanical pressure; their respective merits and demerits; finally concluding with a few unobtrusive suggestions upon the best method of progressing with this operation.

The materials in general use for this purpose consist of wood, cotton, india rubber, etc. I object to wood on account of its being a little too active, as well as the inability to apply it in many cases; for instance, in mouths in which the teeth are in immediate contact, pressed and wedged tightly together, it will be found exceedingly tedious and difficult to push a piece of wood between them, as it is very apt to break or crush in attempting to apply your pushing force. Now, as we have substances not possessing these disadvantages, and yet equal to wood in every other respect, I have abandoned its use, (except in some rare cases.) Of cotton, I would say, my experience pronounces it better in most cases than wood; as being softer and more yielding in character, it more readily accommodates itself to the different widths of the spaces, and consequently can be used with more facility. India-rubber, to my mind, is more objectionable than either wood or cotton, the very qualities which recommend it so highly to the use of some dentists forming the ground-work of my objections, viz., its activity and power. It is altogether for my use, too active, too powerful. The advocates of this article may say they can do more in one hour by its use than they can with any other in two; all of this I frankly admit; and, in so doing, frame my argument against it, considering, as I do, that it does the work too quickly, too effectually: in fact it overdoes it—producing such a sudden and violent separation, that the alveolo-dental periosteums take on a grade of inflammation from which in many cases they never completely recover; leaving even in those instances in which the inflammation does subside, an unpleasant chronic irritability. If india-rubber is used at all, it must be with a degree of caution and care that will never repay for the trouble taken.

I now come to speak of what, in my humble opinion, is the very best material that can be used for wedging teeth apart, and that is ordinary tape, varying in thickness to suit the different-sized spaces. This substance possesses advantages over most others, as far as I am capable of judging, and its application for this purpose was first suggested to me by Prof. J. D. White. The superiority of this article consists in its exerting a gradual and even pressure along the whole lateral surfaces of the teeth, on account of its being the same thickness the entire width of the tape; and, again, having a thin, sharp edge, it is much easier to pass it up between the teeth than any thing else I have mentioned, for cotton is very apt to roll and become lumpy when you attempt to force it into a space that is at all tight; while wood if thin enough to slip up between, will crush and break, and if any thicker will not pass up. I therefore honestly believe, taking everything into consideration, that tape is the best thing we can use to accomplish this end.

In conclusion, permit me to say that, whenever teeth are to be separated by pressure, no matter what means may be used to bring about that separation, the entire operation, from the beginning to the end, should be slowly and gradually performed. I consider it infinitely as important as proper filing; for, even when a tooth is so filed as to eventually cause decay, the disease can be stopped and the preservation of the organ secured by the introduction of a solid, well-finished filling; but once lash the alveolo-dental periosteum up to a high stage of inflammation, and you have brought to birth one of the most harassing and stubborn maladies the dentist has to treat. Here mechanical skill and perfection of manipulation availeth naught; there is now no difficult cavity upon which you have merely to exercise your exquisite fingering and superior instrumental performance, to save the tooth, but you have a disease which baffles, and oftentimes defies, the treatment of the most experienced and scientific—a complaint which, after trying your patience for weeks, and nonplussing every description of remedy, finally terminates with the extraction of the offending part.—*Dental Cosmos.*

